# Supplementary material for: Chloroplast-derived photo-oxidative stress causes changes in H2O2 and EGSH in other subcellular compartments
Source: Plant Physiol. 2021 Jan 6;186(1):125–41. doi: 10.1093/plphys/kiaa095 (PMC8154069; doi:10.1093/plphys/kiaa095)
Supplement: kiaa095_Supplementary_Data [file kiaa095_supplementary_data.pdf]

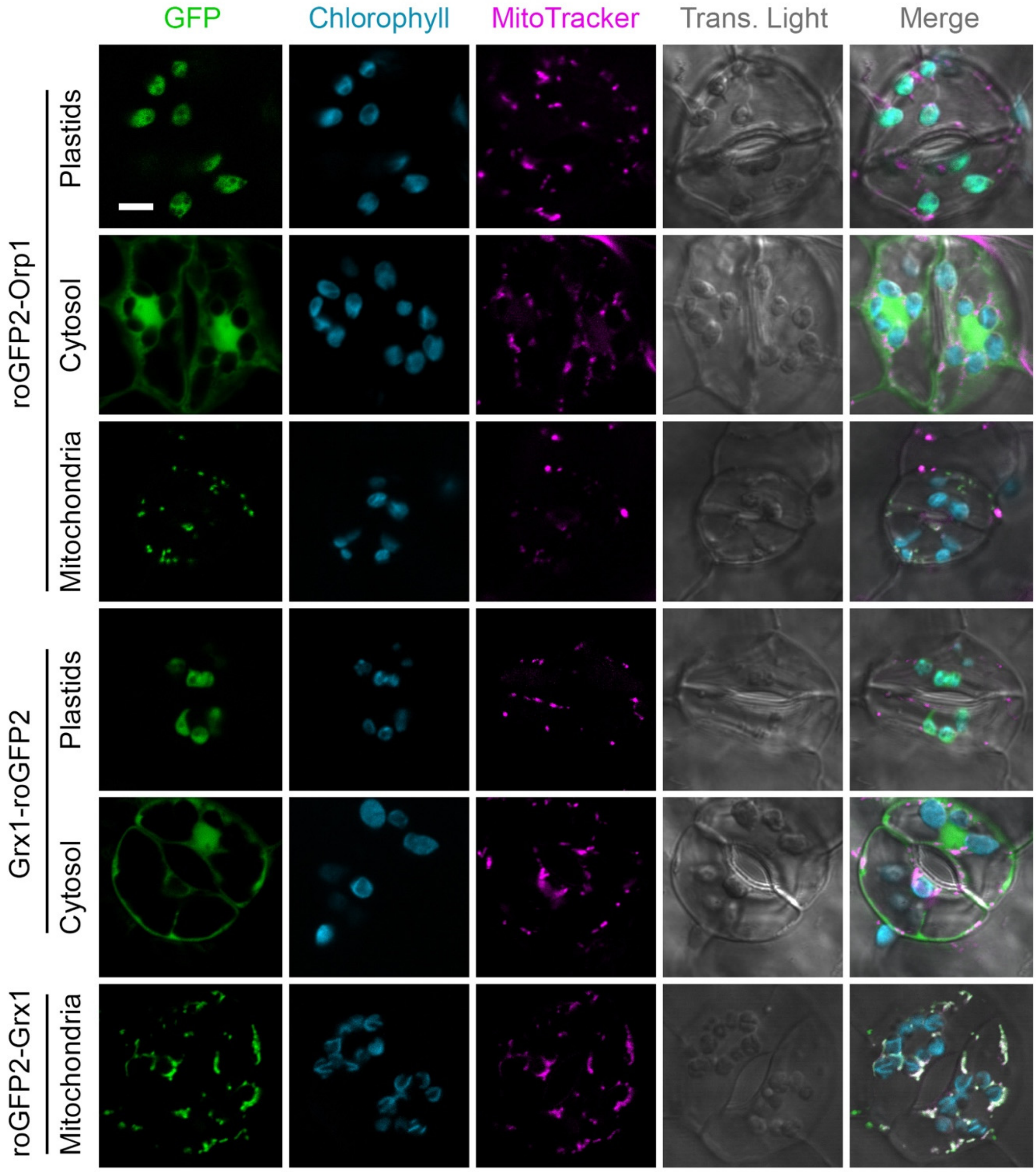

Supplemental Figure S1. Subcellular localization of roGFP2-based probes for the glutathione redox potential (E_{GSH}) and hydrogen peroxide (H_2O_2) in Arabidopsis. A, Confocal microscopy images of stomata from 7-day-old seedlings stably expressing roGFP2-Orp1, Grx1-roGFP2 or roGFP2-Grx1 in the indicated compartments. Images show GFP fluorescence (λex = 488 nm; λem = 505-530 nm), chlorophyll autofluorescence (λex = 488 nm; λem = 650-695 nm) and MitoTracker Orange staining (λex = 543 nm; λem = 570-623 nm). Merge image shows the projection of all three fluorescence channels and the bright field image. Bar, 5 µm.

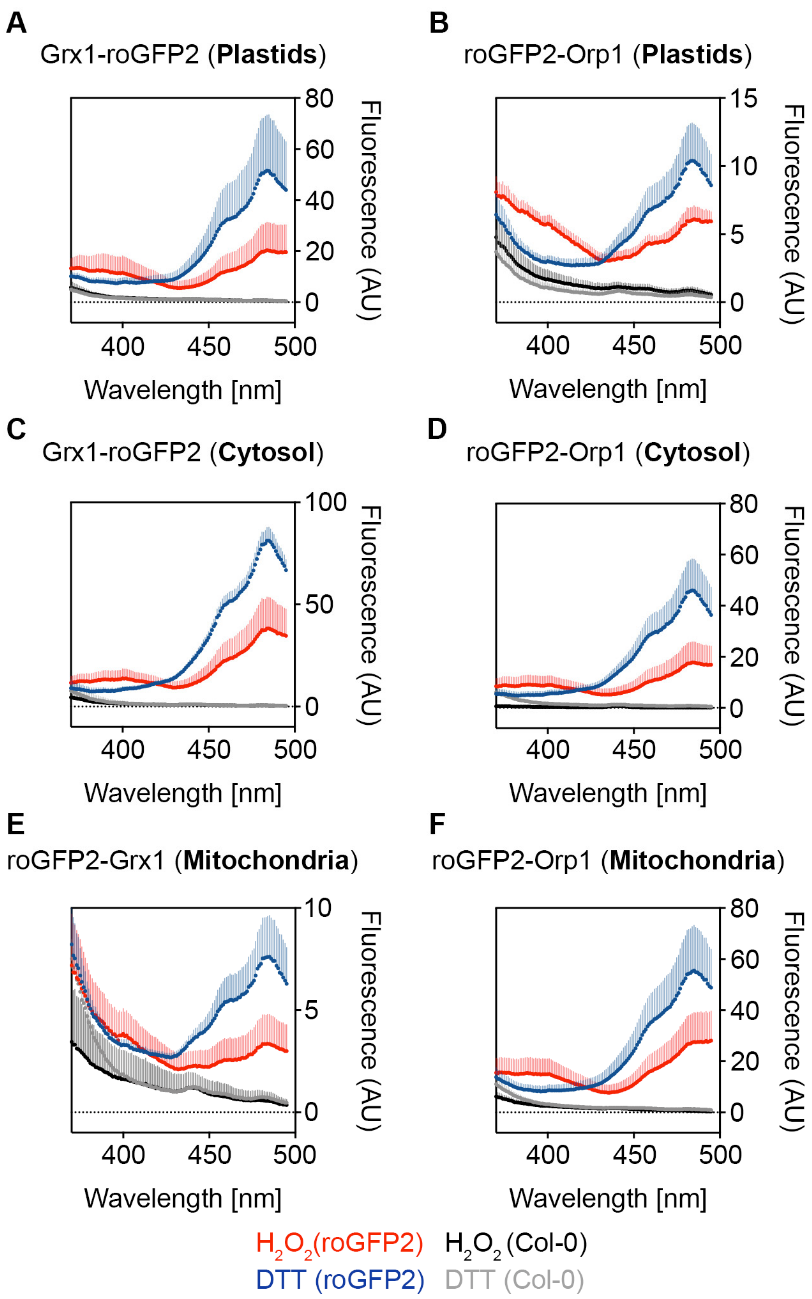

Supplemental Figure S2. Raw fluorescence of Arabidopsis glutathione redox potential (E_{GSH}) and hydrogen peroxide (H_2O_2) sensors lines compared with non-transformed Col-0 plants. A–F, roGFP2 excitation spectra measured in plants expressing Grx1-roGFP2, roGFP2-Grx1 or roGFP2-Orp1 in the indicated subcellular compartments and background fluorescence for non-transformed Col-0 plants. Blue curves show spectra after reduction with 20 mM DTT, whereas red curves indicate spectra after oxidation with 100 mM H_2O_2. Gray and black curves show the same DTT and H_2O_2 treatments on non-transformed Col-0 plants, respectively. Fluorescence excitation spectra were recorded from 7-day-old seedlings at 530±20 nm emission using the same gain for all lines. Mean of arbitrary units of fluorescence (AU) +SD, $n \geq 3$ biological replicates, where each replicate is an independent pool of 4–5 seedlings. Note that overall fluorescence is very low in reporter lines expressing roGFP2-Grx1 in mitochondria, which results is a low signal-to-noise ratio especially at wavelengths < 400 nm.

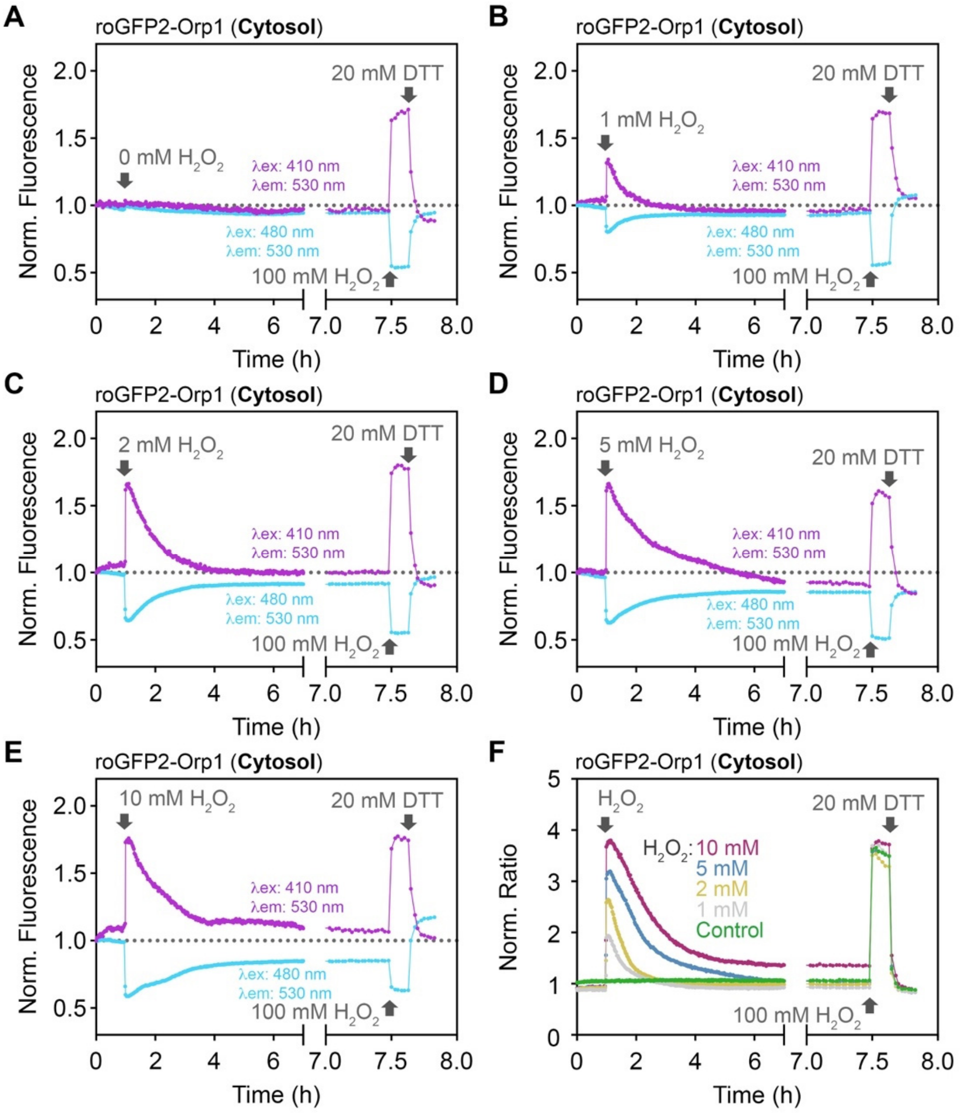

Supplemental Figure S3. Calibration procedure to determine the dynamic range of the sensor exemplified for cytosolic roGFP2-Orp1. A-E, Continuous fluorescence recordings for the independent excitation channels after addition of the indicated concentrations of H_2O_2. H_2O_2 was added after 1 h to final concentrations of 0, 2, 5 or 10 mM. For the 0 mM control only buffer was added. All data are normalized to the initial fluorescence at the start of the experiment, which is also indicated by the dotted line. Mean ratios, $n = 4$ biological replicates. Note that the representative dataset shown in panel E is the same as the time course in Fig. 2E but extended by sensor responses to full oxidation with 100 mM H_2O_2 and full reduction with 20 mM DTT. Pools of 7-day-old seedlings (4-5 per well) expressing roGFP2-Orp1 in the cytosol were placed in a 96-well plate. **F,** The redox state of the sensor was expressed as the normalized fluorescence ratio after sequential excitation of roGFP2 at both excitation wavelengths. At the end of each experiment, plants were incubated in 100 mM H_2O_2 to fully oxidize the sensor, then rinsed twice with imaging buffer and incubated with 20 mM DTT to fully reduce the sensor.

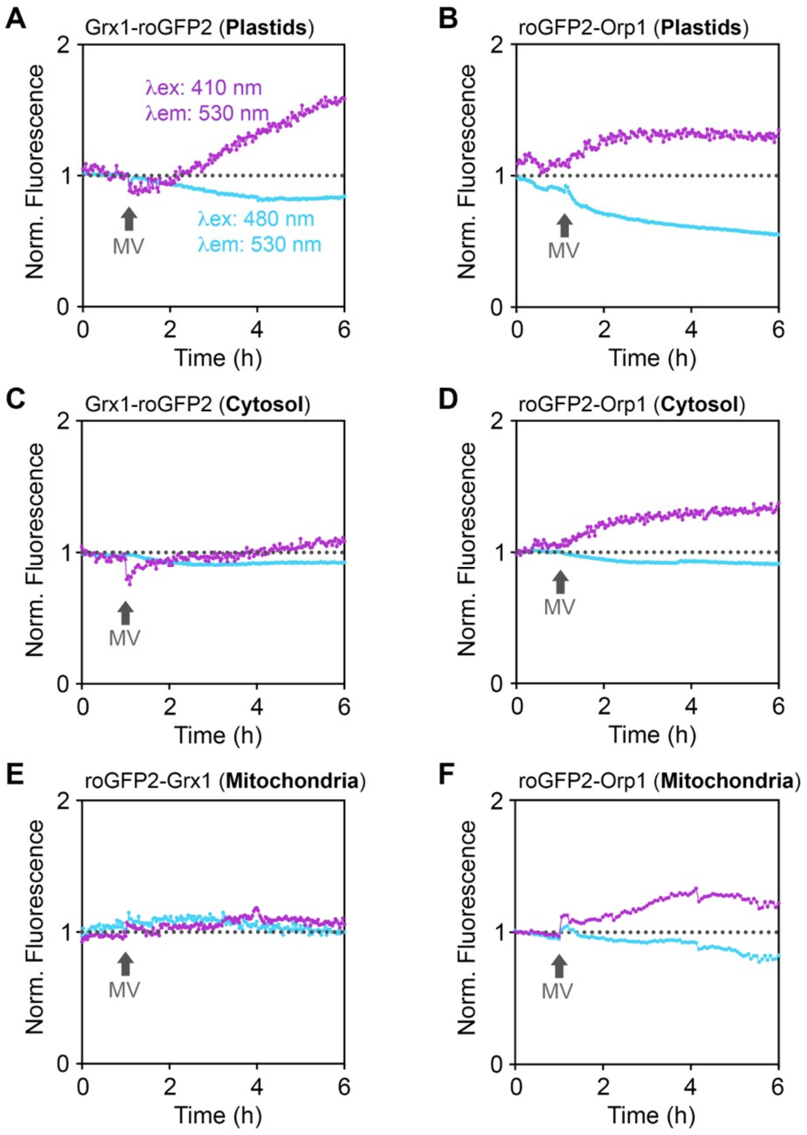

Supplemental Figure S4. Individual excitation channels of roGFP2 fluorescence upon methyl viologen (MV)-induced oxidation *in planta*. A–F, Normalized fluorescence from the two different excitation channels after addition of 100 µM MV. Fluorescence was recorded with the indicated wavelength settings and used to calculate the ratio of Grx1-roGFP2, roGFP-Grx1 or roGFP2-Orp1 sensors, targeted to the cytosol, plastids or mitochondria. Pools of 7-day-old seedlings (4-5 per well) were placed in a 96-well plate. After 1 h, MV was added to a final concentration of 100 µM. Data show the mean fluorescence values of at least 3 biological replicates. The dotted line indicates the initial value of fluorescence.

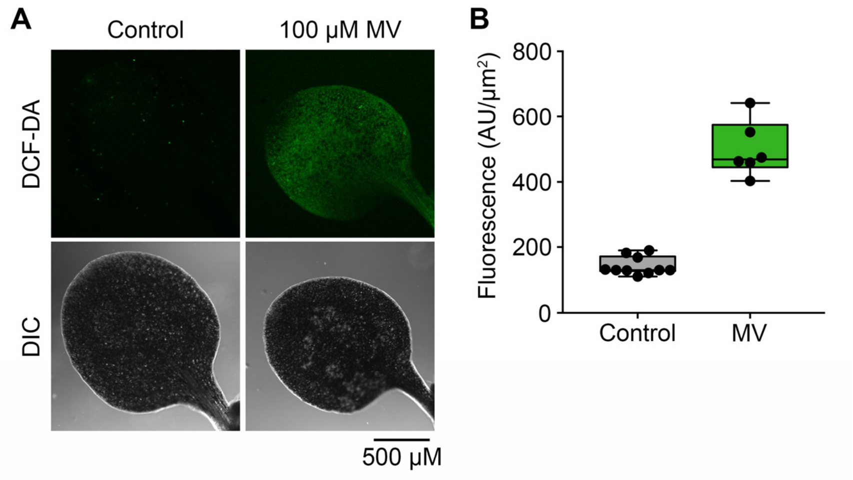

Figure S5. MV treatment causes massive ROS production in leaves. A, Seven-day-old non-fluorescent seedlings were incubated in imaging buffer alone (control) or buffer supplemented with 100 µM MV for 4 h in the light. Subsequently seedlings were stained for 30 minutes with 25 µM DCF-DA and imaged by confocal microscopy exciting the stain at 488 nm and collecting the fluorescence between 505 and 530 nm. **B,** Fluorescence was quantified and expressed as intensity per µm^2 of leaf tissue using FIJI (Schindelin et al., 2012). The box plots show the data from *n* = 6-10 biological replicates, where each replicate is an independent seedling. Box = interquartile range between the lower and upper quartiles, center line = median, whiskers = min and max values.

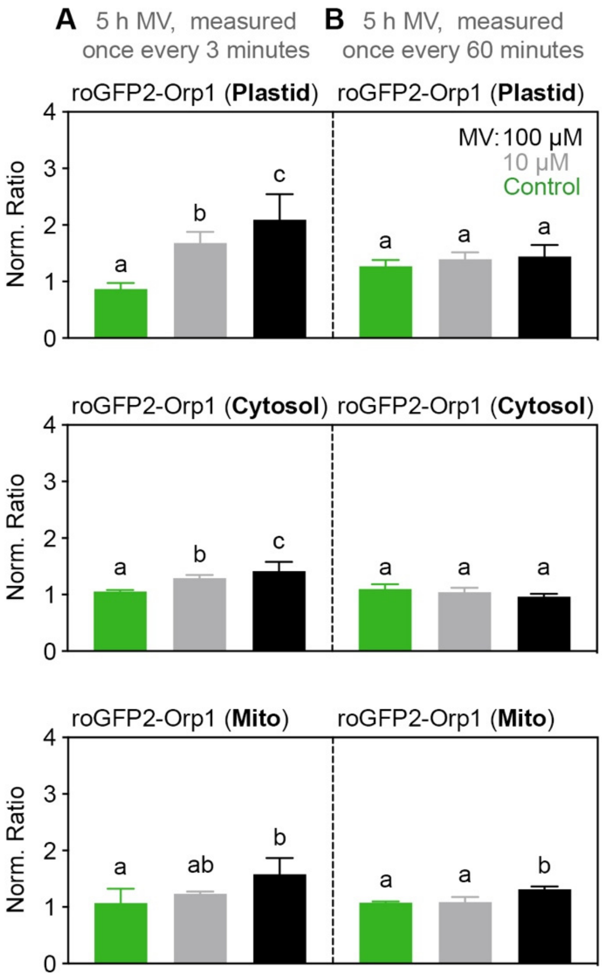

Supplemental Figure S6. Effect of the excitation light on the methyl viologen (MV)-induced oxidation of the roGFP2-Orp1 sensor. A, B, Seven-day-old seedlings stably expressing roGFP2-Orp1 targeted to the plastids, cytosol or mitochondria were placed in a 96-well plate with imaging buffer. After 1 h, MV was added to a final concentration of 10 µM (light gray) or 100 µM (black). In control samples (green), only buffer was added. Normalized ratio values were calculated from the fluorescence recorded once every 3 minutes (A) or once every 60 minutes (B) by sequential excitation of probes at 410 ± 5 nm and 480 ± 5 nm. Fluorescence was always recorded at 530 ± 20 nm. Data indicates the ratio at 5 h after MV addition. Mean ratios + SD, $n \geq 3$ biological replicates, where each replicate is an independent pool of 4-5 seedlings. Different letters indicate statistical differences between ratios after $\log_{10}$ transformation, according to one-way ANOVA with Tukey's multiple comparison test ($P < 0.05$).

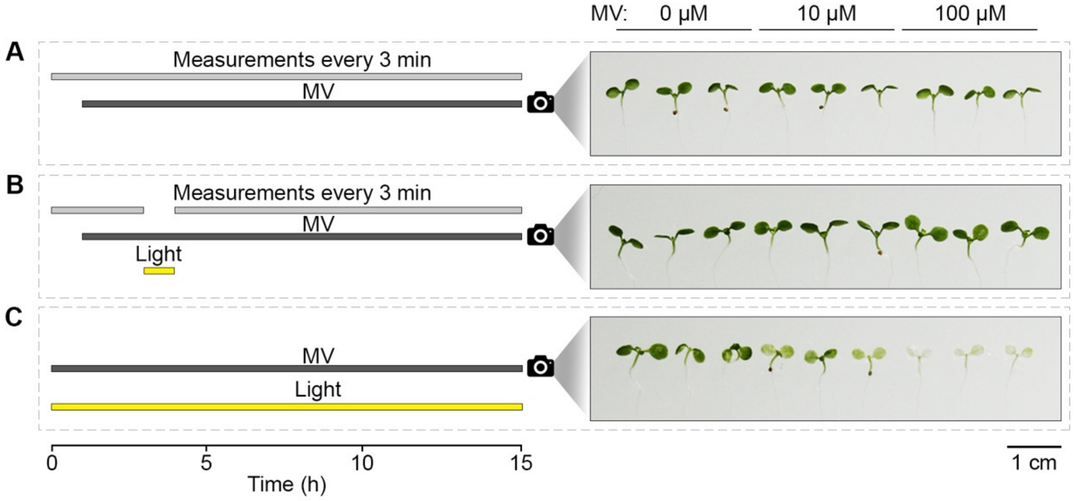

Supplemental Figure S7. Effect of extended treatment of Arabidopsis seedlings with methyl viologen (MV). A-B, Seven–day old Col-0 seedlings were placed in a 96-well plate and immersed in imaging buffer. roGFP2 fluorescence was initially recorded for 1 h in a plate reader every 3 mins. Subsequently, MV was added to final concentrations of 10 or 100 µM (incubation period indicated by black line) and fluorescence recording was continued for 14 hours (A). In a second experiment, the same setup as before was used except that fluorescence recording was interrupted 2 hours after addition of MV for additional illumination of seedlings with constant actinic light at 200 µmol m^{-2} s^{-1} for 1 h. After the 15 h recording seedlings were transferred to agar plates and photographed. **C**, As a control for photo-oxidative damage, seedlings were floated on 0, 10 or 100 µM MV and exposed to constant actinic light at 200 µmol m^{-2} s^{-1} for 15 h and photographed afterwards.

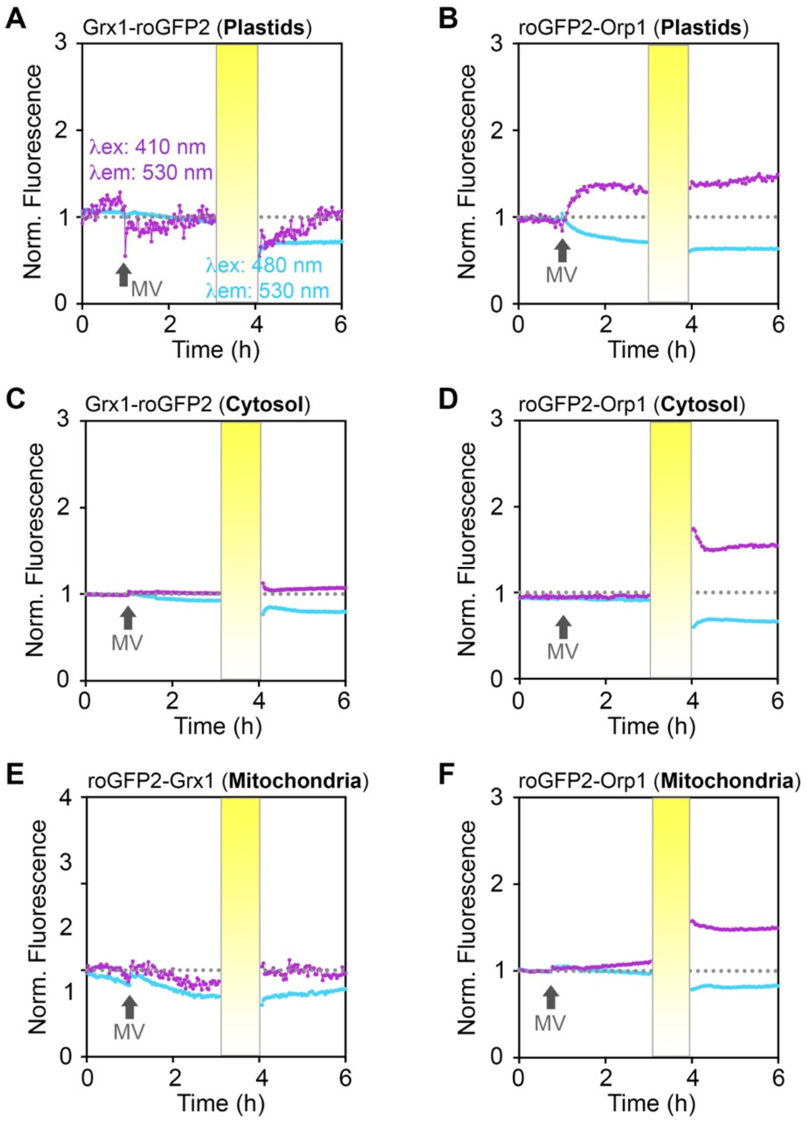

Supplemental Figure S8. Individual excitation channels of roGFP2 fluorescence upon light-enhanced methyl viologen (MV)-induced oxidation *in planta*. A–F, Normalized fluorescence from the independent channels after the addition of 100 µM MV. These channels were used to calculate the ratio of Grx1-roGFP2, roGFP-Grx1 or roGFP2-Orp1 sensors, targeted to the cytosol, plastids or mitochondria, respectively. Pools of 7-day-old seedlings (4-5 per well) were placed in a 96-well plate. After 1 h, MV was added to a final concentration of 100 µM. After 2 h dark incubation, samples were exposed to 1 h of actinic light (200 µmol m^{-2} s^{-1}) before recordings were resumed. Data show the mean fluorescence values of at least 3 biological replicates. The dotted line indicates the initial value of fluorescence.
